# Compulsivity in Alcohol Use Disorder and Obsessive Compulsive Disorder: Implications for Neuromodulation

**DOI:** 10.3389/fnbeh.2019.00070

**Published:** 2019-04-11

**Authors:** Elisabetta Burchi, Nikolaos Makris, Mary R. Lee, Stefano Pallanti, Eric Hollander

**Affiliations:** ^1^Department of Psychiatry and Behavioral Sciences, Albert Einstein College of Medicine, Montefiore Medical Center, Bronx, NY, United States; ^2^Department of Health Sciences, University of Florence, Florence, Italy; ^3^Center for Morphometric Analysis, Massachusetts General Hospital, Harvard Medical School, Boston, MA, United States; ^4^Psychiatry Neuroimaging Laboratory, Brigham and Women’s Hospital, Harvard Medical School, Boston, MA, United States; ^5^Section on Clinical Psychoneuroendocrinology and Neuropsychopharmacology, National Institute on Alcohol Abuse and Alcoholism and National Institute on Drug Abuse, Bethesda, MD, United States; ^6^Department of Psychiatry and Behavioral Sciences, Stanford University Medical Center, Stanford, CA, United States

**Keywords:** alcohol use disorder, compulsivity, obsessive compulsive disorder, habit learning, cognitive control, neuromodulation, transcranial magnetic stimulation

## Abstract

Alcohol use Disorder (AUD) is one of the leading causes of morbidity and mortality worldwide. The progression of the disorder is associated with the development of compulsive alcohol use, which in turn contributes to the high relapse rate and poor longer term functioning reported in most patients, even with treatment. While the Diagnostic and Statistical Manual of Mental Disorders (DSM-5) defines AUD by a cluster of symptoms, parsing its heterogeneous phenotype by domains of behavior such as compulsivity may be a critical step to improve outcomes of this condition. Still, neurobiological underpinnings of compulsivity need to be fully elucidated in AUD in order to better design targeted treatment strategies. In this manuscript, we review and discuss findings supporting common mechanisms between AUD and OCD, dissecting the construct of compulsivity and focusing specifically on characteristic disruptions in habit learning and cognitive control in the two disorders. Finally, neuromodulatory interventions are proposed as a probe to test compulsivity as key pathophysiologic feature of AUD, and as a potential therapy for the subgroup of individuals with compulsive alcohol use, i.e., the more resistant stage of the disorder. This transdiagnostic approach may help to destigmatize the disorder, and suggest potential treatment targets across different conditions.

## Introduction

Alcohol use disorder (AUD), a problematic pattern of alcohol use accompanied by clinically significant impairment or distress (American Psychiatric Association, 2013), is one of the leading causes of morbidity and mortality worldwide (GBD 2016 Alcohol Collaborators, 2018). Globally, with 100.4 million estimated cases, AUD was the most prevalent substance use disorder (SUD) in 2016, with 99.2 million and three quarters of all substance use-attributable disability-adjusted life-years (DALYs). In the United States, 12-month and lifetime AUD prevalence is, respectively, estimated to be up to 14 and 30% of the adult population, with 9.8% of all deaths attributable to acute or chronic alcohol use (World Health Organization, 2018).

Despite there being effective treatments available, only an estimated 21.3 and 34.7% of patients with severe (≥6 DSM criteria) 12-month and lifetime AUD seek treatment in the United States (Grant et al., 2016). Further, high rates of relapse and poor longer term functioning are reported in the minority of patients that get some treatment (Maisto et al., 2018). This is particularly true for the subgroup of individuals with severe AUD (Tuithof et al., 2013), that is also characterized by a longer duration of untreated AUD, a lower level of spontaneous recovery as well as higher rates of psychiatric comorbidity (Grant et al., 2016; Saha et al., 2018). In addition to low rates of treatment utilization, AUD is indeed a heterogeneous disorder (Jellinek, 1960; Moss et al., 2007) and current therapeutic approaches are not developed to address this clinical variation. Hence, the magnitude of the therapeutic effect of the available AUD interventions is, overall, relatively modest (Kranzler and Soyka, 2018).

Next to the need to educate the public and policy makers in order to destigmatize the disorder and increase treatment rates (Keyes et al., 2010), the gaps in research and clinical care call for a shared framework to further characterize this heterogeneous disorder and lead to the development of new therapeutics, ultimately giving the clinician a biologically based roadmap to guide assessment and prioritize treatment (National Institute on Alcohol Abuse and Alcoholism, 2017). In this direction, the National Institute on Alcohol Abuse and Alcoholism (NIAAA) has implemented an Alcohol Addiction Research Domain Criteria (AARDoC) in order to identify specific domains relevant for alcohol addiction (Litten et al., 2015). Recently, incentive salience, negative emotionality and executive function have been proposed as key dimensional domains for the assessment of SUDs and AUD, which map onto the three stage cycle of the development of addiction over time (Koob and Le Moal, 1997; Koob and Volkow, 2016; Kwako et al., 2018). The three stage cycle (Koob and Le Moal, 1997) represents a model that provides an understanding of the development of AUD overtime, and may help organize research in AUD complementing the atemporal RDoC framework. The three stage cycle is consistent with the conceptualization of alcohol addiction as an aberrant form of learning, where alcohol exposure leads in time to alteration in the neurocircuitry underlying stress response, reward and cognitive functioning, all of which ultimately leads to *compulsive* substance use (Wise, 1987; Di Chiara, 1999; Berke and Hyman, 2000; Everitt et al., 2001; Lubman et al., 2004). Hence, compulsive drug seeking has been identified as the central, defining property of alcohol and substance use disorders (Wise and Koob, 2014; NIAAA; National Institute of Drug Abuse). The development of repetitive drug patterns of use, in turn, has been proposed as a main pathophysiologic factor contributing to the high relapse rates characteristic of addiction (Heyman, 2013).

While the Diagnostic and Statistical Manual of Mental Disorders (DSM-5) defines AUD by a cluster of symptoms, parsing its phenotype by domains of behavior, such as compulsivity, may be a critical step to improve outcomes of this condition. On the other hand, neurobiological underpinnings of compulsivity still need to be fully elucidated in addiction -and AUD in particular- in order to design specific targeted treatment strategies.

Compulsivity (Box 1) is a construct that encompasses motor, cognitive, affective and motivational processes. Multiple models have addressed the central question regarding the transition from casual to compulsive drug use over the development of addiction. The traditional hedonic/withdrawal hypothesis of addiction explained compulsive drug use as earlier pleasure seeking and later attempts to avoid unpleasant withdrawal symptoms (Koob and Le Moal, 1997, 2001), but this could not fully explain the high rates of relapse after long period of drug abstinence (Robinson and Berridge, 1993, 2000). More recently this theory has been complemented by models showing how other phenomena take place in the development of addiction, such as incentive sensitization (Robinson and Berridge, 2003), aberrant learning (Robbins and Everitt, 2002), and loss of cognitive control (Jentsch and Taylor, 1999). At the brain connectivity level, these phenomena have demonstrated to map onto limbic-cortico-striatal networks (Ma et al., 2010), with important overlap with neuroimaging findings in OCD (van den Heuvel et al., 2016).

**Box 1. Definitions.**

**Table:** 

**Compulsivity**	The experience of the urge to perform an overt or covert behavior in a stereotyped manner that persists despite lack of goal orientation resulting in not valuable or adverse consequences.
**Habit (Stimulus- Response associations)**	Habitual responses that are directly triggered by stimuli and are defined by insensitivity to their consequences. The S-R associations that mediate habits have been reinforced either by past experiences with reward or by the omission of aversive events.
**Goal directed behavior (Response-Outcome associations)**	Behavior that is mediated by knowledge of the casual relationship between the action and its outcomes and that is performed when the consequences actually constitute a rewarding goal. The goal-directed system exerts control over habits in light of new information.
**Inhibitory Control**	It is a cognitive mechanism that includes exerting control over both goal-directed, reward-seeking (impulsive) and automatic (compulsive) actions.
**Reward**	The subjective salience value of a stimulus that has the potential to induce goal directed behavior.
**Incentive salience**	A form of non-cognitive wanting triggered by reward-related cues and characteristic of the transition from hedonic to habit like compulsive drug seeking; it is explained by conditioned reinforcement of drug-related cues.
**Relapse**	Spontaneous recurrence of a learned behavior (i.e., compulsive drug use) after a given period of extinction.

Following the characterization of the neural basis of compulsivity developed in the field of Obsessive Compulsive Disorder (OCD) research, we discuss the findings supporting common mechanisms between AUD and OCD, focusing on specific aspects of the construct, namely habit learning and cognitive control. Finally, neuromodulatory interventions are proposed as a probe of this hypothesis and as a potential therapy for the subgroup of individuals with AUD who demonstrate compulsive alcohol seeking and use, i.e., the later more severe stage of AUD. In particular, deep transcranial magnetic stimulation (dTMS), recently approved by the Food and Drug Administration to treat compulsivity in OCD, is specifically discussed as a promising non-invasive intervention to treat compulsivity in AUD.

Such a transdiagnostic approach aims to destigmatize AUD, and to help clarify common pathophysiological mechanisms underpinning compulsive behavior across different disorders (Fineberg et al., 2016; Gillan et al., 2016). Consistent with a need for new paths of research in the field of treatments for addictions, the ultimate aim of this manuscript is to highlight and discuss the scientific rationale supporting the development of evidence based treatments for compulsivity in AUD, eventually as a model for other disorders of addiction.

## The Habit Model of Compulsivity

Compulsivity has been defined as a behavioral trait in which actions are persistent and repeated despite adverse consequences (Robbins et al., 2012) (Box 1). An initial hypothesis regarding compulsive behavior in OCD -where compulsivity has traditionally found its paradigmatic expression- purported that it represents a *goal-directed* process associated with a “cognitive bias” or disrupted assignment of value toward available alternative behaviors which are performed to reduce the likelihood that a feared consequence will take place (Salkovskis, 1985; Rachman, 1997). Later, OCD was conceptualized as a disorder of maladaptive *habit learnin*g (Graybiel and Rauch, 2000) on the basis of overlap between the frontostriatal circuits underlying repetitive behavioral habits and OCD (Alexander et al., 1986). Data from subsequent preclinical and clinical studies have actually elucidated the neural basis of habit formation in humans or the transition from goal-directed to habitual behavior as a shift away from signaling in ventral associative frontostriatal circuits comprising ventromedial prefrontal cortex (vmPFC), medial orbitofrontal cortex (mOFC) and caudate, toward that in dorsal sensorimotor frontostriatal circuits, including posterior putamen and premotor cortex (Balleine and O’Doherty, 2010; Gillan and Robbins, 2014). In particular, estimated white matter strength in the vmPFC seeded from the caudate have been found to predict flexible goal-directed action, while estimated white matter tract strength in the premotor cortex seeded from the posterior putamen predicts vulnerability to habitual behavioral control in healthy humans (de Wit S. et al., 2012). Patients with OCD consistently exhibit a cognitive bias toward habit learning over goal-directed behavior (Gillan et al., 2011, 2014; Vaghi et al., 2019) associated with relative higher task-related activity in dorsal vs. ventral striatum and weakened resting state caudate-ventrolateral prefrontal cortex (vlPFC) and putamen-dorsolateral prefrontal cortex (dlPFC) connectivity (Banca et al., 2015; Vaghi et al., 2017). In particular, decreased activity in caudate-prefrontal circuits accompanied by hyperactivation of subthalamic nucleus/putaminal regions have been associated to symptom generation in OCD (Banca et al., 2015). Hyperactivity of putamen preceded deactivation during avoidance/relief events indicating a pivotal role of the putamen in regulation of behavior and habit formation in OCD (Banca et al., 2015). Consistently, task dependent functional connectivity during goal-directed planning showed reduced hypoactivation of the right dlPFC coupled with reduced functional connectivity between this latter and the putamen (Vaghi et al., 2017). Other findings indicate a dissociation between actual behavior and subjectively reported action-outcome knowledge, reinforcing the notion that OCD may be driven by a disrupted goal-directed system over habitual response, rather than by dysfunctional beliefs (Vaghi et al., 2019). This diminished outcome sensitivity has also been associated with diminished caudate-parietal connectivity (Harrison et al., 2013).

Based on these studies, evidence favors the habit hypothesis for compulsivity in OCD: this model posits that rather than goal-directed avoidance behaviors, compulsions are a result of excessive habit formation (Gillan and Robbins, 2014; Burguière et al., 2015). Irrational threat beliefs (obsessions) characteristic of OCD may be a consequence, rather than a cause, of compulsive behavior. In other words, obsessions may be an attempt to resolve the discrepancy between patients’ value attribution and their cognitively inexplicable urge to perform compulsive responses to determined stimuli.

Notably, deficits in goal-directed control and over–reliance on habits are a model of compulsivity that is applicable to other psychiatric disorders where compulsions feature prominently (Hollander, 1993; Hollander and Wong, 1995; Fontenelle et al., 2011; Voon et al., 2015) in particular to drug addiction (Robbins and Everitt, 1999; Gerdeman et al., 2003; Everitt and Robbins, 2005; Hogarth et al., 2013).

## Habit Hypothesis of Compulsivity in AUD

### Compulsivity and Habit Learning in Addiction

Drug addiction has been defined as representing a dysregulation of motivational circuits caused by development of aberrant incentive salience and habit formation, accompanied by compromised executive function over the use of the substance (Koob and Volkow, 2016). Such trajectory correlates with a progression from reward-driven to habit-driven drug-seeking behavior overtime (Koob and Le Moal, 2005; Everitt and Robbins, 2013).

Borrowing from the habit hypothesis of OCD, the experience of “wanting” the drug, progressively detached from the experience of “liking” it (Berridge and Robinson, 2016), may be understood as *post hoc* cognitive rationalizations of a goal-insensitive, stimulus-driven behavior (Gillan and Robbins, 2014). While the expectancy of reward may be crucial to initiate drug self-administration in the early stage of the disorder, the drive toward consumption of the drug triggered by the cue becomes quite indipendent from the expectancy of pleasure in the late stages of the disorder. Thanks to conditioned reinforcement, over time the “wanting” for the drug becomes triggered by the incentive motivational properties of drug cues, independently of a cognitive desire for a declarative goal (Berridge and Robinson, 2016). This shift in phenomenology has been associated with a cascade of neuroadaptations that engage the dorsal striatal habit system over the ventral striatal loop of reward and motivation mediated by phasic dopamine release in the dorsolateral striatum (see for review of clinical studies Volkow and Fowler, 2000; Newlin and Strubler, 2007; Dolan and Dayan, 2013; Grant and Kim, 2014; Koob and Volkow, 2016). In particular, translational studies have shown how a gradual progression from hedonic to habitual and compulsive drug use over time (i.e., a gradual progression from a response that is first dependent and then independent from evaluation of the relationship between action and outcomes eventually becomes a response despite adverse consequences) is associated with (i) habit overlearning and (ii) parallel reduction in goal-directed behaviors and inhibitory control that correspond to a shift in recruitment of ventral to dorsal regions of the striatum (Yin et al., 2004; Belin and Everitt, 2008; Balleine et al., 2009; Lesscher and Vanderschuren, 2012; Willuhn et al., 2012). Intermittent drug–induced dopamine (DA) signaling promotes the ability of drug-paired cues to increase DA levels and recruit striatal-globus pallidal-thalamo-cortical loops that engage the dorsal striatum. This shift from a system dedicated to updating predictions of value (ventral) to a system dedicated to the optimization of reward-related responses (dorsal) augments progression through the addiction cycle and helps explain craving and compulsive drug use (Koob and Volkow, 2016).

### Compulsivity and Habit Learning in AUD

A confluence of preclinical and clinical data strongly supports that the pathogenesis of AUD involves a shift from associative striatum (caudate) to sensorimotor striatum (posterior putamen) in response to alcohol reward responding, likely driven by simultaneous reductions in goal-directed control over actions and increase in habit associations. Learning about response-outcome (R-O) associations has been investigated using instrumental learning paradigms (Balleine and Dickinson, 1998). In instrumental discrimination tasks, which have been developed to distinguish between goal-directed and habit-based learning (de Wit et al., 2007), stimuli (or cues) are congruent, unrelated or incongruent with subsequent outcomes. Whereas performance (or learning) on congruent and control trials can be supported by both the goal-directed (S-R-O) and habitual system (S-O), performance on the incongruent discrimination (in which each pictures functions as a stimulus and an outcome for opposing responses) relies solely on the habit system. The dominance of habitual control over flexible goal-directed responding has traditionally been assessed using revaluation tests, by manipulating outcome value and observing consequent effects on response (Dickinson, 1985). In a typical outcome devaluation methodology (Adams, 1982), after a training phase, the value of the reinforcer of action (O) is typically reduced (extinction phase) affecting internal or external motivational states, and the experimenter assesses if the behavior (R) appropriately updates, in light of this change, measuring the strength of the R-O association. An alcohol addiction model study conducted in rats showed that instrumental alcohol seeking became insensitive to devaluation after 4 weeks of training, mirrored by a shift in control from the dorsomedial striatum to the dorsolateral striatum (Corbit et al., 2012). Another preclinical study using the instrumental learning paradigm showed how alcohol reinforced behavior was less sensitive to devaluation compared to when behavior was reinforced with food, suggesting that alcohol consumption may be particularly susceptible to habit formation (Dickinson et al., 2002). An fMRI clinical study using a cue-reactivity paradigm showed significantly higher cue-induced activation of the dorsal striatum in heavy drinkers compared to social drinkers and higher cue-induced activation of ventral striatum and prefrontal areas in social drinkers compared to heavy drinkers (Vollstädt-Klein et al., 2010). These findings were interpreted as an indirect indication of a shift from ventral to dorsal striatal involvement in the development of AUD, associated with the increasing role of habit–like drug seeking behavior over the course of the disorder. Another clinical study using neuroimaging and learning tasks showed how early abstinent patients presented greater habit formation compared to controls and a progressive shift toward greater goal-directed behaviors with prolonged abstinence (Voon et al., 2015). Another study conducted in AUD patients was the first providing direct behavioral and neurophysiological evidence for an imbalance between goal-directed and habitual control in humans with a substance use disorder (Sjoerds et al., 2013). Subject underwent fMRI during completion of an instrumental learning task characterized by a discrimination learning phase and an outcome-devaluation test phase designed to study the balance between goal-directed and habit learning: patients with AUD compared to healthy controls showed a strong engagement of the neural habit pathway, comprising dorsolateral/posterior parts of the striatum (posterior putamen, caudate tail/body) and a relatively weak engagement of the goal-directed pathway in the vmPFC and dorsomedial/anterior parts of the striatum (caudate head, anterior putamen) during instrumental learning even in the context of AUD-irrelevant stimuli. Moreover, vmPFC activation was negatively associated with AUD duration. Another study investigating the effects of alcohol on devaluation sensitivity for food reward suggested a general effect of alcohol toward habit-formation (Hogarth, 2012).

All these studies give direct (Sjoerds et al., 2013) and indirect (Dickinson et al., 2002; Vollstädt-Klein et al., 2010; Corbit et al., 2012; Hogarth et al., 2012) demonstration of a shift from ventral to dorsal striatal involvement in the development of AUD, associated with an increasing role of habit–like drug seeking behavior over the course of the disorder. Moreover, alcohol consumption has demonstrated to be particularly susceptible to habit formation over other reinforcers (Dickinson et al., 2002) and it has also shown to exert a general direct effect toward habit-formation attenuating goal directed control over action selection (Hogarth, 2012).

Of note, habit circuitry, including the dorsolateral striatum, is also implicated in punishment resistance (Jonkman et al., 2012), characteristic feature of compulsive behaviors, namely behaviors performed in spite of adverse consequences. Indeed, it is important to notice that the construct of “habit” doesn’t completely overlap with the construct of compulsivity, where habitual alcohol use involves behavior that continues despite outcome devaluation, whereas compulsive alcohol use encompasses continued use despite adverse consequences (Hopf and Lesscher, 2014; Marchant et al., 2018). Studies investigating neural correlates of compulsive alcohol seeking using rodent model of aversion-resistant alcohol seeking showed involvement of mPFC, insula and nucleus accumbens (Seif et al., 2013) and hyperactivation of these areas have been found in heavy drinkers as opposed to light drinkers when viewing threat-paired alcohol cues (Grodin et al., 2018).

Next to extensive preclinical evidence suggesting overlap in the striatal regions involved in habitual and compulsive behavior (Everitt and Robbins, 2005; Belin et al., 2009; Pierce and Vanderschuren, 2010; Jonkman et al., 2012; Seif et al., 2013), a recent study conducted on rats for the first time demonstrated the causal importance of the functional recruitment of the anterior dorsolateral striatum (aDLS), a region strongly associated with the consolidation and performance of stimulus-response habits (Yin et al., 2004; Zapata et al., 2010; Corbit et al., 2012), in the switch from controlled to compulsive alcohol use (Giuliano et al., 2019). This study found that individual differences in the reliance of alcohol seeking habits on aDLS dopamine predict and underlie the emergence of compulsive alcohol seeking, providing first evidence that compulsive alcohol seeking stems from an inability to disengage aDLS control over seeking behavior when faced with negative outcomes. This result shows that the maladaptive nature of alcohol seeking in those individuals that become compulsive lays in the rigidity of those aDLS dopamine-dependent habits.

### Compulsivity and Cognitive Control in AUD and OCD

From a neurocognitive point of view, AUD is characterized by an imbalance between overwhelming drive toward alcohol consumption and inability to inhibit alcohol consumption, i.e., a disruption in cognitive control over alcohol use (Baler and Volkow, 2006; Jentsch and Pennington, 2014; Koob and Volkow, 2016). The RdoC domain of cognitive control is defined as a modulatory system that supervises all the activities in the service of goal-directed behavior, when prepotent modes of responding are not adequate to meet the demands of current context. Cognitive (or effortful) control encompasses the ability to select relevant stimuli, inhibit responses influenced by distracting elements, select appropriate responses, monitor the outcome of those responses, and adjust behavior as needed in the face of changing situations.

Despite well documented dysfunctions across multiple cognitive functions (Stavro et al., 2013), deficits in response inhibition have been proposed as the most promising marker of cognitive control impairments measured by behavioral tasks in AUD (Wilcox et al., 2014). Response inhibition, a subdomain of cognitive control and defined as the ability to suppress a pre-potent behavior that is inappropriate or no longer required, has been typically assessed using a range of neuropsychological paradigms including those measuring motor response inhibition (further differentiated into action restraint and action cancelation) and cognitive inhibition (interference control) (Bari and Robbins, 2013). It has been suggested that interference control, action restrain and action cancelation represent early, intermediate and late processes of response inhibition (Sebastian et al., 2013). It has also been proposed that inhibitory control is highest in the action cancelation, making of the Stop Signal Task (SST) the more sensitive task to measure inhibitory control, tapping both impulsivity and compulsivity traits (Smith et al., 2014). In a SST, a “stop” signal appears after the onset of a “go” signal on a subset of trials, requiring the participant to interrupt an ongoing motor response to the go signal that has already been triggered, and the primary dependent measure is the stop-signal reaction time (SSRT). Significant deficits in response inhibition have been observed for all tasks in AUD (Karch et al., 2008; Li et al., 2009) especially using the SST (Smith et al., 2014). Typically, task dependent functional connectivity studies have demonstrated that frontostriatal pathways are critical for response inhibition that is weakened over the progression of AUD: individuals with more severe AUD exhibit impaired connectivity between dorsal striatum and anterior cingulate cortex (ACC), mPFC and mOFC (Courtney et al., 2013; Lee et al., 2013). Studies investigating response inhibition have then further demonstrated the importance of dlPFC-dorsal striatum connectivity for behavioral regulation in AUD especially in the late stage of the disorder (Schulte et al., 2012; Courtney et al., 2013; Müller-Oehring et al., 2013). Moreover, impaired response inhibition in AUD has been related to more intense cue-induced alcohol craving (Papachristou et al., 2013). Importantly, impairments in response inhibition may help differentiate AUD subtypes and predict clinical outcomes (Nigg et al., 2006; Saunders et al., 2008; Schuckit et al., 2012).

In the past several years, deficits in response inhibition have also been described and associated to disrupted functional activations in frontostriatal circuits in OCD involving pre-supplementar motor area (pre-SMA), inferior frontal gyrus, ACC, striatum, thalamus (Maltby et al., 2005; Penadés et al., 2007; Roth et al., 2007; Woolley et al., 2008; Page et al., 2009; de Wit S.J. et al., 2012; van Velzen et al., 2014) and proposed as a neurocognitive endophenotype in OCD (Chamberlain et al., 2007; Menzies et al., 2008; de Wit S.J. et al., 2012). Evidence in both OCD and AUD suggests that disruptions of indirect cortico-striatal-thalamic-cortical (CSTC) pathways may mediate both compulsive behaviors and failure in response inhibition (Table 1) (Aron and Poldrack, 2006; Chambers et al., 2009; Aron, 2011). However, the use of behavioral tasks to dissect cognitive control has some limitations, hampering insights into integrated global brain functioning in non-task related circumstances, which contributes to behavioral variability. Techniques such as *a priori* defined seed-based connectivity help to elucidate how striatal brain areas are integrated into a broader functional network and how that is related to the duration and severity characteristics of alcohol addiction (Schmaal et al., 2013; Müller-Oehring et al., 2015; Galandra et al., 2019). A very recent study coupling resting-state fMRI with an in-depth neuropsychological assessment of the main cognitive domains (Galandra et al., 2019), gives support to the hypotheses that cognitive impairments in AUD (Stavro et al., 2013; Le Berre et al., 2017) are not explained by specific susceptibility of frontal regions to alcohol neurotoxic effects, but rather to dysfunctional connectivity between the cortical (dlPFC and dACC) and subcortical (basal ganglia) nodes of the networks underlying cognitive control on goal-directed behavior. Indeed, while functional connectivity between the basal ganglia and both the dlPFC and dorsal ACC (dACC) nodes was positively related to executive performance in the whole sample, the strength of the very same connections was significantly reduced in patients reflecting the amount of alcohol consumption. The significance of this decrease in connectivity has been investigated by using a graph-theoretical approach, showing that prolonged alcohol dependence is associated with decreased global brain network efficiency and less anterior striatal segregation (Sjoerds et al., 2017). These studies suggest that executive impairment in AUD patients may reflect altered frontostriatal connectivity which underpins top-down modulation of behavior by mediating the switch between automatic and controlled processing. While reward prediction error signal was found intact suggesting proper behavioral options value decoding in the striatum, impaired dlPFC-striatal connectivity has been associated with abnormal processing of financial gains and losses, suggesting that dysfunctional intrinsic connectivity might also underpin defective behavioral learning during task-performance in AUD (Park et al., 2010). Hence, alterations in frontostriatal connectivity underlining impaired learning and decision making may in turn contribute to characteristic poor treatment outcome in AUD. Relapse in AUD has been associated with pronounced atrophy in bilateral OFC, right mPFC and right ACC, impaired connectivity between dorsal striatum and mOFC, and reduced mPFC activation during goal-directed behavior (Grüsser et al., 2004; Beck et al., 2012; Lee et al., 2013; Durazzo and Meyerhoff, 2017; Sebold et al., 2017). On the other hand, other studies have underlined the association between abstinence and normalization in frontostriatal circuits: mOFC-dorsal striatum functional connectivity, impaired in alcohol-dependent patients, showed to increase and normalize in correlation with the duration of abstinence (Lee et al., 2013), and to be associated with a progressive shift toward goal-directed behavior over habitual control (Voon et al., 2015). With progression from short to long-term abstinence, the synchrony within the reward network (subgenual ACC, caudate, nucleus accumbens, and thalamus) and within the executive control network (dlPFC, ACC, and nucleus accumbens) were found to, respectively, decrease and increase progressively (Camchong et al., 2013).

**Table 1:** Fronto-striatal functional connectivity and cognitive control in AUD and OCD.

AUD	OCD
Resting state functional connectivity studies • mOFC-dorsal striatum functional connectivity, increases and normalizes in correlation with the duration of abstinence (Lee et al., 2013). • Synchrony within the reward network (subgenual ACC, caudate, nucleus accumbens, and thalamus) and within the executive control network (dlPFC, ACC, and nucleus accumbens), respectively, decreases and increases with progression from short to long-term abstinence (Camchong et al., 2013). • Decreased global brain network efficiency and less anterior striatal segregation are associated with prolonged alcohol dependence (Sjoerds et al., 2017). • Functional connectivity between the basal ganglia and the dlPFC and dACC nodes is significantly reduced compared to control reflecting the amount of alcohol consumption (Galandra et al., 2019). Task -dependent functional connectivity studies • Abnormal connectivity between the dlPFC and striatum predicts impairments in learning (Park et al., 2010) and response inhibition (Schulte et al., 2012; Courtney et al., 2013; Müller-Oehring et al., 2013). • Synchrony between the PCC and cerebellum is decreased at rest compared to control but increased during a working memory task indicating compensatory networking to achieve normal performance (Chanraud et al., 2011). • Functional connectivity is decreased between PCC and middle cingulate but increased between the midbrain and middle cingulate/MSA and between the midbrain and putamen during Stroop task compared to controls (Schulte et al., 2012). • Functional connectivity between putamen, anterior insula, ACC, and mPFC during SST is decreased compared to controls (Courtney et al., 2013 ).	Resting state functional connectivity studies • Caudate-vlPFC and putamen-dlPFC connectivity is weakened compared to control (Banca et al., 2015). • Reductions in dmPFC and striatum Hyperconnectivity Accompany Successful Treatment (Figee et al., 2013; Dunlop et al., 2016). • Hypoconnectivity within frontoparietal (peaking into dlPFC) and salience network (peaking supramarginal gyrus), and between salience, frontoparietal and default-mode network compared to control (Gürsel et al., 2018). • General dysconnectivity within default-mode (peaking in dmPFC and ACC) and frontoparietal network (peaking in the striatum), as well as between frontoparietal, default-mode, and salience networks compared to control (Gürsel et al., 2018). Task dependent functional connectivity • Functional activations in frontostriatal circuits involving pre-SMA, inferior frontal gyrus, ACC, striatum, thalamus are altered compared to control during response inhibition tasks (Maltby et al., 2005; Roth et al., 2007; Woolley et al., 2008; Page et al., 2009; de Wit S.J. et al., 2012; van Velzen et al., 2014). • Functional connectivity between dlPFC and putamen is reduced during goal directed planning compared to control (Vaghi et al., 2017).

*DS, dorsal striatum; dACC, dorsal anterior cingulate cortex; PCC, posterior cingulate cortex; mPFC, medial prefrontal cortex; mOFC, medial orbito frontal cortex; dlPFC, dorso lateral prefrontal cortex; vlPFC, ventro lateral prefrontal cortex; SST, Stop Signal Task*.

Meta-analysis of seed-based resting-state fMRI studies in OCD found consistent hypoconnectivity within frontoparietal (peaking into dlPFC) and salience network (peaking supramarginal gyrus), and between salience, frontoparietal and default-mode network (Gürsel et al., 2018); consistent general dysconnectivity was found within default-mode (peaking in dmPFC and ACC) and frontoparietal network (peaking in the striatum), as well as between frontoparietal, default-mode, and salience networks (Gürsel et al., 2018).

These findings match perfectly with the structures comprising the CSTS circuits, namely OFC, ACC, vmPFC, striatum, and thalamus (Pauls et al., 2014).

While the CSTC loop hypothesis has dominated the OCD literature over the last decades (Alexander et al., 1986), as the nature of integration has become apparent for the processing of information in the striatum, such that information is carried in “spirals,” rather than isolated “loops” (Milad and Rauch, 2012) more attention has been paid to potential “hubs” in the neurocircuitry thought to underlie OCD.

**FIGURE 1:**
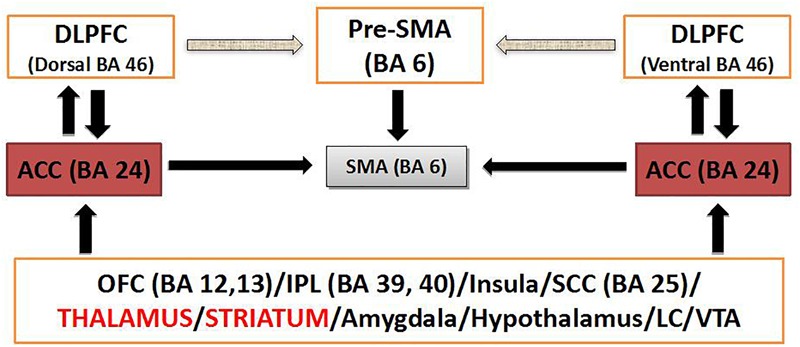
Schematic diagram of dACC circuitry implicated in modulation of cognitive control. BA, Brodmann’s area; DLPFC, dosolateral prefrontal cortex; IPL, inferior parietal lobule; LC, locus coeruleus; SMA, supplementary motor area; VTA, ventral tegmental area.

The dorsal ACC has been proposed as a connective hub of cognitive control (Shackman et al., 2011) in a ideal position to receive sensory input and act on that information via downstream motor regulation (Figure 1 and Box 2). Proposing cognitive control as a main domain in OCD pathophysiology, dACC dysfunction and consequent aberrant cognitive control signal specification has been proposed to drive the pursuit of tasks that do not accord to long-term goal, underlying the core pathophysiology of OCD (McGovern and Sheth, 2017).

**Box 2.**

Cognitive control and the potentiality of targeting the dACC. Within the RDOC construct of cognitive control and, more specifically the subdomain of response inhibition/response selection, the neural network of the anterior cingulo-insular or salience network (aCIN) seems to be critically relevant (Downar et al., 2016). The dACC stands midway along a hierarchy of medial prefrontal regions that guide goal selection, thought selection and action selection based on internal drives as opposed to external cues. The dACC is active when choosing freely between to different cognitive tasks and during voluntary internally cued modulation of emotional state (Downar et al., 2016). The anterior cingulate cortex (ACC) interfaces attention, executive function, drive, affect and motor control. Convergent data from neuroimaging, neuropsychological, genetics, and neurochemical studies have implicated, bilaterally, dysfunction of dorsolateral prefrontal and anterior cingulate cortical structures, which constitute the cortical arm of the frontostriatal network (caudate and putamen being its subcortical counterpart) driving executive function (Solanto, 1984; Castellanos, 1997; Zametkin and Liotta, 1998; Giedd et al., 2001; Seidman et al., 2006; Makris et al., 2007), in cognitive dyscontrol. The ACC (Brodmann’s area 24 or BA 24) is an important regulator of other cortical and subcortical brain regions as well, and its disconnection appears to be consistent with an executive dysfunction. The ACC is connected with other cortical areas ipsilaterally and controlaterally as well as with subcortical structures. The topographic organization of these axonal connections is precise within the subcortical white matter and architectonically arranged within the overlaying cortical layers of the ACC (Figure 1). This mesh of connections converging to and diverging from the neuronal bodies of the ACC constitutes a central networking node within the overall connectional map of the brain crucial for the interface of drive, emotion, cognition and motricity (Paus, 2001) as well as for the modulation of cognitive control (Cohen et al., 2000). Short (U-fibers) and medium range association fibers connect BA 24 with adjacent cortical areas. The more complex long association fibers belong to three discrete fiber bundles, namely the cingulum bundle (CB) (Mufson and Pandya, 1984), the uncinate fascicle (UF) (Petrides and Pandya, 1988) and the extreme capsule (EmC) (Petrides and Pandya, 1988). Through these three fiber pathways the anterior cingulate cortex is connected in a bidirectional fashion as follows (Schmahmann and Pandya, 2007). The CB connects BA 24 with rostral posterior cingulate BA 23, paracingulate BA 32 and orbital frontal BA 14, 12, and 11 as well as prefrontal BA 8 and 6 (Baleydier and Mauguiere, 1980). Via the UF, BA 24 connects with subcallosal BA 25 and basal forebrain regions as well as the amygdala and the perirhinal cortex. The EmC provides connections with the dysgranular insula and the insular proisocortex. Striatal fibers by virtue of the subcallosal fascicle of Muratoff connect BA 24 to the caudate’s head and, through the external capsule, with the putamen and claustrum. Thalamic connections are with anterior, the medial dorsal as well as the midline thalamic nuclei. Other connections are with the parahippocampus, the hippocampus, the zona incerta and the pons as well as with the locus coeruleus and the ventral tegmental area (Paus, 2001). Motor connections are with the supplementary motor area, the motor cortex and the spinal cord (He et al., 1995). Chronic failure of the ACC network may have profound implications contributing to deficit of cognitive control. Convergent evidence from fMRI (Carter et al., 1998, 1999; Botvinick et al., 1999; Cohen et al., 2000) and evoked potential (Gehring and Knight, 2000) experimentation in humans suggest that ACC is associated with monitoring of conflict and modulation of cognitive control as well as modulation of allocation of attention in real time. As illustrated in Figure 1, interactions between the dorsolateral prefrontal (DLPFC), inferior parietal lobule (IPL), orbital frontal cortex (FOC), amygdala and brainstem centers such as the locus coeruleus or the ventral tegmental area, enable the ACC to integrate sensitive information in real time to monitor conflict of competitive cognitive tasks, modulate cognitive control and produce balanced behavior (Yeterian and Pandya, 1985; Cohen et al., 2000). It appears that alterations in the ACC and its associated frontostriatal network (primarily the caudate nucleus and putamen being its subcortical counterpart), which are driving executive function, are critical in executive dysfunction. Therefore, neuromodulatory interventions seem to be an important therapeutic means to modulate ACC function and restore balance in behavior when cognitive control is lost in such conditions as AUD.

Functional connectivity studies have suggested that cognitive and behavioral alterations observed in AUD might reflect functional imbalances within a CTST loop involving the key nodes of the reward (Park et al., 2010; Camchong et al., 2013; Müller-Oehring et al., 2013), salience (Sullivan et al., 2013; Zhu et al., 2017) and executive networks (Weiland et al., 2014; Zhu et al., 2017; Galandra et al., 2019), namely striatum, dlPFC, and dACC. Activity of the cingulate cortex is also emerging as a marker of subsequent alcohol relapse (De Ridder et al., 2011; Zakiniaeiz et al., 2016).

## Neuromodulatory Strategies to Target Compulsivity in AUD

### State of Art in the Treatment of AUD

A variety of therapeutical approaches are currently available for AUD, such as behavioral treatments, medications and mutual-support groups (NIAAA treatment Navigator page). While, due to the anonymous nature of mutual-support groups, it is difficult to determine their success rates compared with those led by health professionals, cognitive behavioral therapy (CBT), motivational enhancement therapy (MET), marital and family counseling and other brief interventions have demonstrated to be beneficial in AUD. In addition to behavioral treatments, there are currently three FDA approved medications for the post withdrawal maintenance of alcohol abstinence, namely disulfiram, naltrexone, and acamprosate (Kranzler and Soyka, 2018). Disulfiram works negatively reinforcing aversion toward alcohol by inhibiting the enzyme acetaldehyde dehydrogenase and resulting in unpleasant effects when combined with alcohol (Mutschler et al., 2016). Naltrexone is an opiate antagonist that is hypothesized to work buffering the endorphin-mediated rewarding effects of alcohol (Mason et al., 2002). Acamprosate was thought to reduce craving for alcohol by acting as GABA agonist and modulator of glutamate NMDA activity, both disrupted by chronic alcohol use (Mason et al., 2002) and recent evidence suggests that its anti-relapse effects may act via calcium (Spanagel et al., 2014). However, given the broad distribution of neurotransmitter systems in the brain, it is particularly difficult to have a targeted action on neural circuits using pharmacotherapeutics.

Although a review of pharmacotherapy is out of the scope of this paper and mechanisms of action have to be fully elucidated (Koob and Mason, 2016), the available medications for AUD mainly try to reduce the positive reinforcing properties of alcohol or the negative reinforcing aspects of chronic alcohol use by relieving craving (Heilig et al., 2010).

Research has provided evidence that the propensity to engage in drug/alcohol-seeking is determined by the expected value and probability of getting the drug (Hogarth, 2012). Whereas the overall propensity to engage in a goal-directed choice is determined by the expected outcome value of the outcome, in habit learning the capacity of cues to elicit this choice is determined by the expected probability of getting the outcome. Hence, in habit learning, devaluing the outcome does not affect the response. In other words, the actual treatments may have partial efficacy because they may work on tonic (expected alcohol value), but not on cue-elicited phasic DA signaling (Hogarth et al., 2014). In order to improve treatment outcome, new interventions should be developed to directly target inflexible, habitual cue-elicited drug-seeking behavior.

### Neuromodulation to Treat Compulsivity in AUD

Following the conceptual framework outlined above, neuromodulatory interventions able to selectively target frontostriatal circuitries may held therapeutic promise for the treatment of compulsive alcohol seeking in AUD. Promisingly, effective neuromodulatory interventions for compulsive symptoms have been associated with normalization in connectivity and restored behavioral control from the striatum to prefrontal cortical regions (Mian et al., 2010; Figee et al., 2013; Dunlop et al., 2016).

Whereas transcranial direct current stimulation (tDCS) needs more investigation for the treatment of compulsivity given the lack of sham controlled studies, repetitive Transcranial Magnetic Stimulation (rTMS) (Box 2) as non-invasive and relatively site specific, has been the most studied neuromodulation technique in the treatment of compulsive behavior in both AUD and OCD (Campbell et al., 2018; Shivakumar et al., 2019). As the time of writing, there have been 10 studies probing rTMS as a tool to change alcohol consumption and explore the associated neurocircuit changes in AUD (Table 2). Different outcome measures were used to assess the impact of different stimulation protocols on relapse, wanting of the drug (“craving”), cognitive control and associated funtional connectivity (Table 2). Traditional rTMS studies targeting the right dlPFC had some success at reducing alcohol craving (Mishra et al., 2010, 2015; Herremans et al., 2015) in accordance with the results obtained by a sham controlled tDCS study applied to the same area (Boggio et al., 2008). To date, the majority of interventional rTMS studies in OCD have been conducted stimulating the dlPFC as well, with mixed results. On the other hand, the most recent meta-analysis to assess whether the effectiveness of rTMS in improving OCD symptoms is moderated by its application over different cortical targets revealed that rTMS applied over the SMA yields greater improvements than rTMS applied over the dlPFC or OFC (Rehn et al., 2018). This therapeutic effect has been attributed to the normalization of hyperactive orbito-fronto-striatal circuits induced by low frequency-rTMS (LF-rTMS) (Mantovani et al., 2010). The SMA plays a central role in motor planning and response-inhibition (de Wit S.J. et al., 2012) and has extensive connections to regions involved in cognitive and emotional processes. Studies suggest that hyperactivity in this area may be associated with deficient inhibitory control over repetitive behaviors (Oliveri et al., 2003), thus making it an attractive target for the inhibitory effects of LF-rTMS. The efficacy of neurosurgical treatments for the treatment of resistant OCD patients suggests other promising deep targets for treating compulsivity. Stereotactic lesions in the dACC (cingulotomy) have shown long term efficay in the treatment of refractory OCD with average full response of 41% (Sheth et al., 2013; Brown et al., 2016). Based on good results with stereotactic ablation, Deep Brain Stimulation (DBS) has been explored and DBS of anterior and ventral capsule, ventral striatum or subthalamic nucleus (STN) has shown a global percentage of responders of 60.0% with long-term efficacy (Bais et al., 2014). Stimulation of STN has been reported to decrease OFC and mPFC metabolism, as well as ACC activity, while stimulation of striatal areas has been associated with decrease in OFC and subgenual ACC activity (Alonso et al., 2015).

**Table 2:** Studies using rTMS as a tool to change alcohol consumption.

Study	Subjects	Study Design	Site and protocol of stimulation	Outcome measures	Results
Mishra et al., 2010	45 patients	Single blind, sham controlled study.	10 Hz, total 10 sessions over the right DLPFC.	Alcohol craving questionnaire.	Significant reduction in craving in the active group compared to sham. Moderate effect size.
De Ridder et al., 2011	1 patient with severe untractable craving.		1–35 Hz stimulations over 3 months targeting the dACC using a double coil.	EEG beta activity and functional connectivity (BOLD activity).	Craving was associated with EEG beta activity and connectivity between dACC and PCC that disappeared after successful rTMS. Cue-induced worsening of craving promoted activation ofACC,vmPFC,PCC, nucleus accumbens that disappeared on fMRI following successful rTMS. Relapseassociated with recurrence of ACC and PCC EEG activity in gamma band.
Höppner et al., 2011	19 female detoxified patients.	Blind sham controlled study.	10 sessions of HF (20 Hz) rTMS applied to the left DLPFC.	Obsessive Compulsive Drinking scale.	No significant differences between sham and active group.
Herremans et al., 2013	29 detoxified patients.	Single blind sham-controlled crossover study.	1 session of HF (20 Hz) rTMS applied to the right DLPFC.	Commission errors, mean reaction times, intra-individual reaction time variability during a Go-NoGo task. Obsessive Compulsive Drinking Scale.	Only the active stimulation reduced the intra individual reaction time variablity, suggesting that even one session stabilizes cognitive performance. No effects of stimulation on craving.
Ceccanti et al., 2015	18 patients	Randomized double blind sham controlled pilot study.	10 sessions of 20 Hz dTMS (H coil) applied to the mPFC.	Cortisolemia Prolactinemia. Craving visual analogic scale.	dTMS significantly reduced cortisolemia and prolactinemia suggesting a rebalancing of the dopamine-cortisol equilibrium during alcohol withdrawal. Craving decreased in treated patients, as well as mean number of drinks/per day.
Herremans et al., 2015	26 detoxified patients.	Open label study.	15 sessions of F rTMS applied to the right DLPFC HF-rTMS over 4 days.	Ten-point Likert scales (for cue-induced craving). Alcohol Urge Questionnaire the Obsessive Compulsive Drinking Scale (for general craving).	General craving significantly decreased after the 15 sessions. Cue-induced alcohol craving was not altered. The craving neurocircuit was not directly affected during an alcohol related exposure, but instead the attentional network was influenced.
Mishra et al., 2015	20 detoxified male patients	Single-blind, parallel-group, active-comparator rTMS study.	10 sessions of 10 Hz rTMS over either right or left DLPFC.	Alcohol Craving Questionnaire (ACQ-NOW).	Significant reduction in craving scores in patients receiving either right or left rTMS with large effect size. No difference in anticraving efficacy between the two groups.
Herremans et al., 2016	19 detoxified patients.	Open label study.	14 sessions of HF (20 Hz) rTMS applied to the right DLPFC spread over 3 days. Before and after stimulation, patients were confronted with a block and event related alcoholic cue exposure paradigm.	Relapse (consumption of any amount of alcohol within 4 weeks after the stimulation) functional connectivity (BOLD activation).	Abstainers (6) compared to patients who had relapsed (16) had higher dACC activation at baseline, but only during blocked cue-exposure paradigm suggesting higher baseline dACC as a protective factor for relpase. The lower the baseline dACC activation, the more dACC activity was increased after HF-rTMS treatment dACC (Rate dependent change in ACC).
Addolorato et al., 2017	11 male patients	Double blind sham controlled study.	12 dTMS sessions 10 Hz over bilateral DLPFC.	Dopamine transporter (DAT) availability by Single Photon Emission Computed Tomography (SPECT) in the striatum. Obsessive Compulsive Drinking Scale (OCDS).	After 1 month of rTMS sessions, striatal DAT availability decreased in the REAL group, being no longer different from HC, whereas it remained unchanged in SHAM patients a reduction of alcohol intake and an increase of the number of abstinence days was found only in the REAL rTMS. In particular alcohol craving decreased in both REAL and SHAM patients, although changes did not reach statistical significance.
Hanlon et al., 2017	24 patients	Single blind sham controlled crossover study.	1 session of real or sham cTBS over the left frontal pole.	Evoked BOLD signal.	Real cTBS significantly decreased evoked BOLD signal in OFC, insula, and lateral sensorimotor cortex.

Despite the limited number of cases examining DBS in patients with AUD, DBS of the nucleus accumbens has shown long term treatment benefit, reducing alcohol craving for up to 8 years associated with modulation of anterior mid-cingulate cortex functioning and cognitive control (Kuhn et al., 2011; Müller et al., 2016). In light of the striking results obtained with DBS of striatal areas and the evidence of aDLS dopamine dependent alcohol seeking (Giuliano et al., 2019), the recent development of new coils designed to target deep cerebral areas with rTMS, may be particularly interesting, avoiding the complications and adverse events related to invasive techniques and possibly gaining some of their therapeutic advantages. Indeed, there is already evidence that cortical stimulation with deep TMS (dTMS) may modulate sub-cortical striatal activity in AUD (Addolorato et al., 2017). While rTMS proved to indirectly modulate the insula and the ACC by stimulating the dlPFC (Nahas et al., 2001) or the frontal pole (Hanlon et al., 2017), H coil design series now promise to target these deeper structures directly, with greater effectiveness. A recent big randomized study investigated the efficacy of dTMS in OCD (Carmi et al., 2018): after provocation of symptoms, 99 treatment resistant OCD patients were treated with either high frequency (HF) or sham dTMS over the mPFC and ACC for 6 weeks and 29 sessions. Despite the lack of neuroimaging, this study conveys relevant results in terms of response rate (38,1%) and maintenance (4 weeks follow up) and to date it is the largest TMS controlled study ever conducted in OCD which led to FDA approval to market the dTMS system for treatment of OCD.

In addition, the mPFC and ACC have been associated with initiation of compulsive behavior in OCD (Rauch et al., 1994; Adler et al., 2000; Viol et al., 2019), they have also been individuated as core regions activating during alcohol cue processing (Heinz et al., 2009; Zakiniaeiz et al., 2016;Grodin et al., 2018; Hanlon et al., 2018) and may be considered as promising new targets for dTMS in AUD (Ceccanti et al., 2015; Herremans et al., 2016).

## Summary and Perspective

Findings suggest that later stages of AUD may be better conceptualized as a disorder characterized by compulsive features, namely overreliance on stimulus-driven habits at the expense of flexible, goal-directed action, leading to frequent and persistent substance use despite serious negative consequences (Belin and Everitt, 2008; Fontenelle et al., 2011). In other words, although alcohol seeking is initially a goal-directed behavior consolidated by operant conditioning, in which alcohol is sought for its rewarding effect, it becomes ultimately a maladaptive optimized response elicited by alcohol-associated stimuli, characterized by over-active striatal habit forming circuitries coupled with lack of sufficient inhibitory control (Chamberlain et al., 2005; Vanderschuren and Everitt, 2005; Menzies et al., 2008; Smith et al., 2014). As seen in OCD, there is a lack of extinction of obsessions (Lovibond et al., 2009), in AUD there is disruption in extinction learning of ethanol-seeking behavior with persistency of the behavior overtime despite adverse consequences (Gass et al., 2017; Grodin et al., 2018). Development of compulsivity may thus explain part of the treatment resistance and relapse in AUD (Sinha et al., 2011; Courtney et al., 2013; Lee et al., 2013). Despite the residual presence of a reward component in driving the behavior, craving for alcohol in the late stages of the disorder is comparable to a compulsion, in phenomenology – in the emergence of urges in response to alcohol-related cues and the inability to resist them- and in neurocircuitry.

The field of cognitive neuroscience can provide measures that are a reflection of the underlying neurobiology, and may eventually inform treatment selection. Disruption in inhibitory control has been proposed as endophenotype and pathophysiologic factor in the development of OCD and AUD (Chamberlain et al., 2007; Yücel and Lubman, 2007; Menzies et al., 2008; de Wit S.J. et al., 2012; Jentsch and Pennington, 2014; Leeman et al., 2014; Wilcox et al., 2014; Gillan et al., 2016). In particular, impaired response inhibition is associated with severity, duration of illness, impaired goal-directed planning and reduced frontostriatal connectivity in AUD. These findings suggest that in AUD response inhibition may be used as a useful marker of cognitive impairment, disruption in connectivity between frontal (dlPFC and dACC) and striatal nodes, as well as a potential treatment target. Recent studies have demonstrated how brain stimulation techniques may affect response inhibition, suggesting how non-invasive neuromulation may be particularly promising in order to develop treatments whose effect in drinking outcome are mediated by improvement in cognitive control (Nakamura-Palacios et al., 2012; Nardone et al., 2012). Future work is still needed to clearly identify the most reliable and valid markers for the deficits, and the degree to which deficits or changes in cognitive control moderate or mediate response to particular treatments in AUD. Recent findings point to the hypotheses that cognitive impairments in AUD are related to dysfunctional connectivity between cortical (dlPFC and dACC) and subcortical nodes (basal ganglia). Thus, studies aimed to characterize instrinsic resting state connectivity and hubs in the reward, salience and executive networks in AUD patients may be particularly useful for the development of new treatment strategies.

Treatment with rTMS is still in its infancy but is a promising tool for developing effective and viable circuit-specific treatment strategies in AUD. The current evidence suggests that targeting inflexible seeking responses may offer a therapeutic strategy to promote abstinence and prevent relapse in AUD. The results of the available studies, in accordance with recent network models of addiction (Dunlop et al., 2017) seems to point to the potential of mPFC and dACC (Box 3 and Figure 1) as TMS targets for the treatment of compulsive alcohol seeking in AUD. The available findings suggest that those areas may be pivotal in order to enhance cortico-striatal-thalamic connectivity and capacity for response selection/inhibition, with the potential to act on the two core aspects of incentive salience and habit learning (Box 3 and Figure 1). Future efforts to improve outcome for rTMS in AUD will likely benefit from rigorous manipulation of the cognitive state during neuromodulation.

**Box 3.**

Repetitive transcranial magnetic stimulation as a tool for personalized psychiatry. In rTMS, single TMS pulses are delivered at various frequency (typically 1–20 Hz) in either a fixed or bursting pattern from 600 to 4000 pulses per session. There is general agreement that low frequency (LF) stimulation (e.g., 1 Hz) causes long term depression of cortical excitability, whereas higher frequency (HF) stimulation (e.g., 10–20 HZ) induces long term potentiation of cortical excitability. These effects can be achieved through teta burst stimulation (TBS). With continuous TBS (cTBS), three pulse bursts at 50 Hz are applied at a frequency of 5 Hz. In most protocols, this cycle continues until 600 pulses have been delivered (20 s). For intermittent TBS (iTBS), bursts are applied at the same rate (five groups of three pulse bursts per second) for 2 s, followed by an 8-s pause. In most protocols this 10-s cycle occurs until 600 pulses have been delivered (190 s). When performed over the primary motor cortex, 600 pulses of cTBS inhibit cortical excitability, whereas 600 pulses of iTBS amplify cortical excitability (Huang et al., 2005). The advantage of TBS protocols is that effect sizes comparable to fixed frequency protocols can be achieved significantly faster (1–2 min versus 20–30 min). The spatial resolution and penetration depth of a TMS pulse depend on the coil. Typical figure-of-eight coil affects approximately 10 cm^2^ of cortical surface, while H shape coil design approximately 100 cm^2^. Most flat coil designs have penetration depths from 1 to 2 cm, whereas the H-coil designs has higer depths of 2–3 cm (Deng et al., 2014). The introduction of H-coils has offered the opportunity of non-invasively modulate activity in brain targets that previously were accessible only by neurosurgial procedures. The combined use of neuronavigation and neuroimaging with rTMS, makes of this latter a feasible therapeutic tool for personalized psychiatry.

## Conclusion

Despite effective treatments that are available in AUD, there remain high rates of relapse and poor long term functioning even in those patients who get therapy. We describe evidence supporting the role of compulsivity (persistent use despite adverse consequences), in the development of AUD resistant to approved interventions. Following a transdiagnostical approach and using OCD as comparison, we highlight features of compulsivity in AUD, showing how phenotypical similarities between the two disorders involve overlapping pathophysiological mechanisms. After identifying compulsivity as a promising and neglected target domain for new treatment approaches in AUD, we discuss neuromodulatory interventions in order to improve recovery in AUD.

For disorders, such as AUD, whose development relies on learning, rTMS gives the unique opportunity to non-invasively act on target neurocircuits with the best space-time resolution. The possibility to couple rTMS with specific tasks designed to activate specific associated circuits allows unique opportunity for personalized therapy. The current evidence seems to point to the potential of mPFC and dACC as TMS targets for the treatment of compulsive alcohol seeking in AUD. Further longitudinal studies combining neuromodulation with neuroimaging and neurocognitive measures are needed to shed light on additional mechanisms underlying rTMS effects in AUD patients, and to test the hypotheses that targeting compulsivity, the habit system and inhibitory control, normalizing fronto-striatal function may convey treatment benefit in AUD.

In conclusion, the conceptualization of AUD within a dimensional and learning framework opens new opportunities for research and clinical management:

• It underlines the importance of reducing the duration of untreated illness in order to prevent development of compulsivity, associated to resistance to treatment and relapse.
• It guides the implementation of a stepped care approach, that considers different diagnostic and treatment strategies in relation to the stages of AUD, underlying the importance of assessing and treating compulsivity.
• It supports recovery as a realistic goal, based on the opportunity of modulating neuroplasticity.
• It supports the implementation of studies aimed to investigate neuromodulation as a promising treatment strategy.

## Author Contributions

EB and EH contributed to the conceptualization of the manuscript. EB wrote the manuscript. NM wrote Box 3. All authors contributed to manuscript revisions, and read and approved the submitted version.

## Conflict of Interest Statement

EH received a research grant to study dTMS in OCD from BrainsWay. The remaining authors declare that the research was conducted in the absence of any commercial or financial relationships that could be construed as a potential conflict of interest.
